# On Wines in Relation to the Amount of Phosphorus They Contain

**Published:** 1855-11

**Authors:** 


On Wines in Relation to the Amount of Phosphorus they Contain.—Dr. Kletzinsky, as the result of an extensive comparative analysis of different wines, draws the following conclusions as to their phosphatic contents:

1. Phosphate of magnesia is a constant constituent part of wine, independently of locality, goodness or age.
2. The quantity of phosphoric salts undergoes considerable variations.
3. These hold a very direct and certain relation to the goodness of the wine; so that their quantitative determination is, perhaps, a better measure of this than is that of the alcohol or extractive.

The therapeutical employment of wines has hitherto been guided by the amount of extractive, alcohol and free acid they contain: but this additional element, owing to the agency of phosphorus in the reparation of the nervous, muscular, and osseous structures, is of no less importance in the direction of our choice. Re-convalescence in typhus, or exhausting exudative processes, the so-called adynamiæ, and the crowd of chronic affections that, chemically speaking, depend upon a poverty of phosphorus, exhibiting themselves, sooner or later, in the form of rickets, scrofula or neuralgia, indicate the employment of wines containing a large proportion of phosphorus. In vain do we endeavor to relieve diseases characterized by an excessive consumption phosphorus, (*Phosphoro-phthisis*,) by the administration of calcined oyster shells, bone-ash, the osteolithes, and phosphorites of the mineralogists, or any other combination of inorganic phosphorus with lime. They pass through the alimentary canal, unabsorbed and unassimilated, the possibility of assimilation depending upon organic combinations.

The author gives a tabular view of the results of his analysis of the several wines, exhibiting their per centage of alcohol, extractive and phosphates. As far as the last are concerned we find that Tokay contains 5 per 1000, Menes and Malaga, 4; Maderia and Sherry, $3\frac{3}{4}$; Santorino, $3\frac{1}{3}$; Cyprus, $3\frac{1}{4}$; Cape, $2\frac{1}{8}$; Château-Lafitte, 2; Rhenish Wines, $1\frac{1}{2}$; Champagne, $1\frac{1}{4}$.—*London Lan.*

